# Combining cohorts of prospectively collected and linked data to improve health after release from prison

**DOI:** 10.23889/ijpds.v9i5.2682

**Published:** 2024-09-18

**Authors:** Matthew Legge, Lindsay Pearce, Craig Cummings, Michael Curtis, Natalia Yee, Kimberlie Dean, David Preen, Mark Stoove, Stuart Kinner

**Affiliations:** 1 Justice Health Group, Curtin School of Population Health and enAble Institute, Curtin University; 2 School for Population and Global Health, University of Western Australia; 3 Justice Health Group, Centre for Adolescent Health, Murdoch Children’s Research Institute; 4 Disease Elimination, Burnet Institute; 5 National Drug Research Institute, Curtin University; 6 Justice Health and Forensic Mental Health Network, New South Wales Health; 7 Discipline of Psychiatry and Mental Health, School of Clinical Medicine, Faculty of Medicine University of New South Wales; 8 Australian Research Centre in Sex, Health and Society, La Trobe University; 9 Melbourne School of Population and Global Health, University of Melbourne

## Background

While people who experience incarceration have remarkably poor health profiles, undertaking research to inform evidence-based responses is complicated by difficulties of recruiting people in prison; high rates of socioeconomic marginalisation, study attrition; and legislative and financial barriers to linked data research. There are many advantages to pooling data from multiple studies involving people who experience incarceration, including greater statistical power and geographic and participant heterogeneity. However, there are also challenges that need to be addressed.

## Methods

We combined four prospective cohort studies of adults released from prisons in four Australian states. Pre-release interviews, and validated screening assessments, were linked to primary care, medicine dispensing, hospital, alcohol and other drug treatment services, ambulatory mental health, ambulance, corrective services, and death records. Data were harmonised by team members reviewing variable definitions and categories within subject domains.

## Results

The combined cohort consists of 4,232 adults, including 1,544 Indigenous people and 905 women, with a median age of 31 years. Data linkage will enable a median of 9.3 years of prospective follow-up after release from incarceration. Differences in data structures, coding systems between and within datasets, changes over time, and grouping of records belonging to the same event were also addressed.

## Conclusion

This combined multi-site cohort study is an example of a complex, policy-oriented data linkage project. It was developed to underpin evidence-based, culturally appropriate interventions, health policy and service development for people who were incarcerated. This presentation will discuss the processes and pitfalls experienced while building a multi-sectoral, multi-jurisdictional data linkage project.

